# Prioritising measures and interventions to strengthen research reproducibility: a Delphi consultation study

**DOI:** 10.1186/s41073-026-00217-y

**Published:** 2026-07-03

**Authors:** Dora Pejdo, Ivan Buljan, Ana Marušić

**Affiliations:** 1 Department of Research in Biomedicine and Health and Center for Evidence-Based Medicine, School of Medicine, University of Split, Split, Croatia; 2 Faculty of Humanities and Social Sciences, University of Split, Split, Croatia

**Keywords:** Prioritisation, Reproducibility, Experts, Transparency, Interventions

## Abstract

**Background:**

Research reproducibility – the ability of others to independently verify scientific findings by following the same methods and data – is a fundamental aspect of research integrity. When this fails, trust in evidence is undermined and research resources are wasted. Reproducibility measures describe the standards that define reproducible research, and reproducibility interventions are the actions used to improve those standards. There is little agreement on which practices and initiatives should be prioritised for implementation in practice. This study aimed to establish expert consensus on the key measures and interventions that should be prioritised to strengthen research reproducibility.

**Methods:**

We conducted a Delphi consensus study as part of the EU Horizon Europe iRISE project. Experts from five stakeholder groups (researchers, editors, publishers, funders, and policymakers) evaluated reproducibility measures and interventions identified through prior literature mapping across two online survey rounds, followed by a final virtual consensus panel. Items were rated on a 10-point Likert scale, with consensus defined a priori as agreement by at least 70% of panellists assigning high-priority scores, corresponding to scores of eight to ten on the Likert scale.

**Results:**

Seventy-three panellists from 34 countries participated in the first round, with high retention rates in the subsequent round. Consensus was achieved on eight reproducibility measures and six reproducibility interventions in the first round. Prioritised measures included methodological quality, reporting quality, code and data availability and reuse, computational reproducibility, transparency of research plan, reproducible workflow practices, trial registration, and materials availability and reuse. Prioritised interventions included data management training, data quality checks/feedback, statistical training, data sharing policy/guidelines, protocol/trial registration, and reproducible code/analysis training.

**Conclusions:**

The prioritised measures and interventions provide a structured foundation for improving research reproducibility across disciplines. The findings can inform the development of institutional training curricula in data management and statistical methods, policies supporting data sharing and trial registration, and future empirical evaluation of these practices across research contexts.

**Supplementary Information:**

The online version contains supplementary material available at 10.1186/s41073-026-00217-y.

## Background

Research reproducibility is a fundamental aspect of research integrity – the commitment to conducting and reporting research honestly, transparently, and rigorously – as the reproducibility concept ensures that scientific findings can be independently verified and trusted. It also promotes transparency by requiring researchers to clearly report their methods, data, and analyses, allowing others to confirm or challenge the results [1]. Ensuring the reproducibility and integrity of research findings is fundamental to scientific progress and public trust in science [1, 2]. Over the past decade, the topic of reproducibility has received increasing attention from researchers, institutions, and funders, as it underpins the credibility and utility of scientific knowledge [3].

Failures to reproduce key findings have been reported across multiple disciplines, raising concerns about the reliability of published evidence and the efficiency of research investments [4]. Persistent issues such as inadequate methodological transparency, selective reporting, and limited data sharing have contributed to what is often termed a reproducibility crisis across disciplines [3, 5]. These challenges highlight systemic issues such as ineffective reporting standards, selective publication practices, and a lack of transparency in data and methods [6, 7]. Addressing these problems requires coordinated and evidence-informed actions across the entire research ecosystem rather than isolated efforts focused on individual researchers or institutions, such as promoting preregistration, distributing badges, sharing data, training in research methods, etc. [2, 8]. The issue requires a more systemic approach incorporating all stakeholders involved in research [9].

Efforts to address the issue have included development of reporting guidelines, open data and preregistration platforms, journal-level data sharing policies, and funder driven transparency initiatives [10]. Despite increasing awareness, however, these efforts to enhance research reproducibility remain fragmented, and the implementation of evidence-based practices is inconsistent across research domains [10, 11]. Within the framework of the EU Horizon Europe project “Improving Research Reproducibility” (iRISE), these challenges were addressed by identifying, assessing, and promoting effective interventions that strengthen reproducibility and research integrity across the scientific ecosystem [12]. A systematic mapping of existing measures and interventions aimed at improving reproducibility across diverse scientific disciplines identified a range of initiatives from training programmes and reporting guidelines to data sharing policies and preregistration platforms [13] but also emphasised a limited consensus regarding which interventions should be prioritised for implementation by researchers, institutions, funders, and policymakers [14, 15].

To address this gap, a Delphi consensus study was conducted to prioritise reproducibility interventions and measures identified in literature mapping. In this study, *reproducibility measures* refer to aspects of research practice that describe the conditions needed for a study to be reproducible, such as methodological quality, reporting quality, preregistration, data and code availability, and others [13]. *Reproducibility interventions* refer to the actions that support these practices, including training programmes, protocol or trial registration procedures, data-management support, the use of open repositories, and others [13]. Together, these measures and interventions outline what reproducible research requires and what can be done to promote it.

## Methods

### Study design and rationale

The Delphi method is particularly suited to complex topics where empirical evidence is limited, but expert opinion can provide structured insight [16, 17]. Its iterative format, anonymity, and controlled feedback facilitate joint expert judgment while minimising bias. The present paper reports the results of the Delphi consultation, which aimed to establish expert-driven priorities for improving research reproducibility across disciplines.

The Delphi process was conducted throughout two online rating rounds followed by a final virtual consensus panel. Each round included both quantitative and qualitative information to ensure that participants’ priorities and rationales were captured.

The protocol was created prior to data collection and preregistered on the Open Science Framework (OSF) [18]. The Survey Monkey platform (SurveyMonkey Inc., San Mateo, USA) was used to conduct survey rounds, and the final consensus meeting took place online via the Microsoft Teams platform (Microsoft Cooperation, Redmond, United States).

The study report adheres to the CREDES checklist for open reporting of Delphi studies [19], see additional file 3.

### Expert panel

A purposive sampling strategy was employed to recruit experts from a cohort of approximately 300 potential experts identified through Stakeholder Forum of the iRISE consortium and associated Horizon Europe projects on research reproducibility (e.g., TIER2 and OSIRIS), as well as through institutional and organisational contacts with experience in reproducibility-related initiatives, participants of the World Conference on Research Integrity, and members of professional networks associated with research reproducibility and integrity. Invitations were distributed via email until the target number of participants was reached. Inclusion criteria for the survey rounds required recognised experience in research reproducibility or closely related topics, such as open science, research integrity, and data sharing, evidenced by publications, completed projects, or active professional engagement in the field. Participants were also required to have no conflict of interest and willingness to participate. The panel included representatives from five key stakeholder groups: researchers, editors, publishers, funders, and policymakers. Within each group, experts were recruited from a range of disciplines and institutional contexts, reflecting the intentionally cross-disciplinary scope of the study.

The final Delphi consensus panel comprised eight participants, representing the principal stakeholder groups involved in research reproducibility. The panel members were purposively selected according to their expertise, experience, and availability to ensure that the final panel group provided balanced representations of the main stakeholder groups and their opinions.

### Delphi process

Before the first Delphi round, all invited participants received an information document detailing the study’s purpose, objectives, and procedures, along with an informed consent form. The document presented the definitions of reproducibility measures and reproducibility interventions (see additional file 1), the rating procedure, and the criteria for determining consensus. The participants were informed about the use of a 10-point Likert scale for scoring and were encouraged to provide written comments to explain their decisions. Approximately one week before round 1, the participants were provided with access to the list of reproducibility measures and interventions identified through a Systematic Online Living Evidence Summary (SOLES) – a continuously updated, curated synthesis of the latest evidence, akin to a scoping review, conducted as a preceding step within the iRISE project. Its use was a requirement of the grant agreement, ensuring methodological coherence across the project’s interconnected work packages [13]. The consent forms were submitted electronically when each participant received their unique code, which was used throughout two rounds, to follow the responses. Only the participants who provided informed consent were included in the Delphi process.

### Prevention of bias

The anonymity of the responses was maintained during the rating rounds to reduce the influence of dominant individuals and to encourage honest expression of opinions. The iterative design with controlled feedback allowed participants to reconsider their ratings in light of group opinions without peer pressure. To mitigate order effects and response fatigue, the presentation order of reproducibility measures and interventions was randomised in each round. Reminder emails sent midway through and again two days before closure to encourage timely completion and reduce nonresponse bias. The research team monitored possible attrition across rounds, and participant representation was assessed to ensure balanced input from the stakeholder groups.

### Description of methods and procedure

The Delphi consultation was implemented in three stages: a preparatory phase, two online rating rounds, and a final virtual consensus panel. Invitations were sent from 23 October 2024. Round 1 was conducted from 29 October to 12 December 2024; the extended duration was necessary to reach the target number of participants. Following analysis of Round 1 data and preparation of anonymised feedback, Round 2 was conducted from 7 to 21 January 2025. The final consensus panel took place on 2 May 2025, with the interval between Round 2 and the panel allowing for scheduling coordination among participants from multiple countries. The preparatory phase involved developing the survey instruments. The survey was structured around the list of reproducibility measures and interventions identified through systematic mapping of literature under the same project. The full list of reproducibility measures and interventions evaluated is presented in the Table 1 (see Additional file 1 for definitions). Survey was reviewed by the research team and colleagues within the iRISE project prior to distribution.

**Table 1:** Reproducibility measures and interventions evaluated in the Delphi round 1

**Reproducibility measures (n = 14)**
Code and data availability and re-use^*^
Computational reproducibility^*^
Materials availability and re-use^*^
Methodological quality^*^
Transparency of research plan^*^
Absence of publication bias
Reporting quality^*^
Reproducible workflow practices^*^
Transparency of contributions
Transparency of evaluation
Transparency of funding
Transparency of interest
Trial registration*
Type I/II error reduction
**Reproducibility interventions (n = 27)**
Reporting guidelines and checklists
Blockchain technology
Centralized sharing platform
Code quality checks/feedback
Code sharing policy/guidelines
Computational reproducibility checks/feedback^†^
Data access policies/agreements^†^
Data quality checks/feedback^*^
Data sharing policy/guidelines^*^
Data sharing statements
Data management training^*^
Documentation system (e.g. electronic lab notebooks)
Materials sharing policy/guidelines
Mentoring/role model
Online sharing platform
Open-access publication
Open Science tools (Open Code badges, Open Data badges, Open Materials badges, OpenStats, Open Science Framework)
Preregistration badges
Protocol/trial registration^*^
Registered reports
Reporting quality checks/feedback (e.g. Penelope.ai)
Reproducible code/analysis training^*^
Reproducible coding environment
Choice of statistical plan (e.g. high-powered studies, multi-center setting, conservative *p*-values)
Statistical training^*^
Critical assessment training
Workflow standardization

^*^Item reached consensus and was confirmed by the final consensus panel

^†^Item reconsidered by the final consensus panel but not included in the final priority list

In the first round, the panellists were asked to rate each reproducibility measure and intervention on a 10-point Likert scale (see Table 2), with the possibility of explaining their choices by leaving a comment.

**Table 2:** Consensus criteria and decision rules applied across Delphi rounds

Score range	≥ 70% of participants	Classification	Action
8—10	Yes	High priority	Reaching priority list
4—7	Yes	No consensus	Carrying forward to the next round for re-evaluation
1—3	Yes	Low priority	Excluding from further rounds

^*^Scores represent participant ratings on a 10-point Likert scale. Consensus was defined as agreement by ≥ 70% of participants

In the second round, panellists were provided with anonymised feedback from round 1, including summary statistics, items that reached priority lists, and aggregated comments for each item brought forward to round 2, to allow reflection and potential adjustment of their previous scores. The same consensus criteria were applied. The reminder emails were sent midway and two days before closure to maximise participation.

The list of reproducibility measures and interventions is available as an additional file 2.

The final round consisted of an online meeting with eight selected panellists (two researchers, two editors, two publishers, one funder, and one policymaker). During this session, the participants revisited the highest-scoring interventions that had not reached consensus in previous rounds. The panel then had the task of reviewing the ranking order of the two prioritised lists.

### Consensus definition

Table 2 presents the consensus criteria and decision rules across Delphi rounds. A 70% threshold is commonly used in Delphi studies to represent group consensus [20, 21]. The same consensus rules were applied to both Delphi rounds. In the second round, the interventions and reproducibility measures that were classified as *high priority* in the first round were listed.

In the final Delphi panel, any interventions or reproducibility measures that were close to but below the predefined consensus threshold were reconsidered to ensure that potentially important items were not excluded. The purpose of the final panel was to provide an opportunity for structured discussion of these borderline items and to allow panellists to review and confirm the ordering of the prioritised lists after completing all survey rounds. Qualitative comments collected throughout the process were used to identify areas of agreement and uncertainty and to refine the presentation of items between rounds.

### Interpretation and processing of results

The quantitative data from each Delphi round were exported from the SurveyMonkey platform and analysed descriptively at the panel level. For each reproducibility measure and intervention, weighted averages and the proportion of participants assigning scores within the predefined ranges (1–3, 4–7, 8–10) were calculated to determine whether the 70% consensus threshold was achieved. Weighted average scores correspond to the mean of participants’ ratings on the scale, which are calculated by weighting each response option by the number of respondents selecting it. Items meeting or exceeding this threshold were classified as either prioritised, discarded, or carried forward, in accordance with the study protocol. Kendall’s coefficient of concordance was calculated separately for each round of the Delphi method to assess the agreement among panellists in their prioritisation ratings. Kendall’s W ranges from 0 (no agreement) to 1 (complete agreement), with values below 0.3 typically indicating low agreement, values of approximately 0.3–0.5 indicating moderate agreement, and higher values reflecting stronger consensus [22].

Qualitative comments accompanying each rating were examined via summative content analysis to identify key themes, areas of disagreement, or requests for clarification [23]. This involved identifying frequently recurring topics across participant feedback, quantifying their occurrence where relevant, and interpreting their contextual meaning to explain scoring patterns. This process allowed the integration of qualitative reasoning with quantitative results, clarifying why certain items achieved or failed to achieve consensus across rounds. This analysis informed both the refinement of items between rounds and the interpretation of consensus patterns. These insights were used to refine the wording and presentation of items in subsequent rounds and to inform the discussion during the final consensus panel. During the final round, both the statistical outcomes and the qualitative feedback were considered jointly to decide on the prioritised lists and ensure that the final results reflected balanced input from all the stakeholder groups.

Weighted averages and consensus calculations were performed in a spreadsheet, whereas qualitative content analysis was conducted manually by one researcher (DP) and independently reviewed by another researcher (IB) to ensure completeness and accuracy. AM provided methodological supervision throughout process, and the three-author team was considered sufficient given the experience and scope of the study and the support of the iRISE project consortium in participant identification.

### External validation

A separate external validation phase was not conducted, as the Delphi process itself incorporated iterative internal validation through structured feedback and repeated evaluation across rounds. Each subsequent round served to verify and refine the results of the preceding round by providing panellists with anonymised summaries of group responses and comments. Prior to the first round, all study materials, including the list of reproducibility measures and interventions and the survey instruments, were reviewed by the research team to ensure clarity, relevance, and consistency with the study objectives. In addition, the final consensus panel functions as a validation step, allowing participants from diverse stakeholder groups to review, confirm, and endorse the final prioritised lists.

### Ethical considerations

This study received ethical approval from the Ethics Committee of the University of Split School of Medicine (Reg. No. *2181-198-03-24-24-0067*). All participants provided informed consent prior to taking part in the study. All the data were collected, processed, and stored in accordance with GDPR requirements and institutional data-protection procedures (General Data Protection Regulation, 2016).

## Results

### Participant characteristics and response rates

Among the approximately 300 invited experts, 73 participants from 34 countries agreed to participate and completed round 1. These countries spanned five regions: Europe (n = 20 countries), Asia (n = 6), Africa (n = 4), the Americas (n = 3), and Oceania (n = 1). Round 2 included 66 participants (90.4% retention). The panel represented five stakeholder groups: researchers, editors, publishers, funders and policymakers. Most participants held a PhD or postdoctoral qualification (n = 61), with the remainder holding a university degree (n = 12). Regarding experience in the field, 41.4% of participants reported more than 20 years of experience, 30.1% reported 11—20 years, 20.5% reported 6—10 years, and 8.2% reported fewer than five years, reflecting an overall highly experiences panel. Disciplines represented included health sciences (n = 27), social sciences (n = 26), natural sciences (n = 15), humanities and arts (n = 9), and engineering and technology (n = 4), with one participant from agricultural and veterinary sciences. The final consensus panel comprised eight participants (two researchers, two editors, two publishers, one funder, and one policymaker), purposively selected to ensure balanced stakeholder representation.

### Quantitative findings

Among the 14 reproducibility measures, 8 reached the priority list, and the scoring of 6 items within the predefined range of 4–7 was carried forward to round 2 for re-evaluation. Among the 27 interventions, 6 reached the priority list, and 21 items were carried out to the second round for re-evaluation. In the second round, no new items reached the priority list. There was a low-to-moderate level of agreement across items, with agreement increasing in the second round (Kendall’s W = 0.257 and W = 0.396, respectively). All the items retained high participation rates across rounds, and no items were excluded because of a lack of consensus or disengagement.

Table 3 presents the eight reproducibility measures and six interventions that met the predefined threshold for inclusion in the priority lists. The weighted averages for these items ranged from 9.03 to 8.16 for the measures and from 8.52 to 8.11 for the interventions, each achieving at least 70% consensus. In contrast, items that did not reach the ≥ 70% consensus threshold had weighted averages of 7.00–7.88 for measures and 5.84–7.97 for interventions, reflecting conceptual support tempered by feasibility, enforcement, or cross-disciplinary applicability concerns.

**Table 3:** Prioritised reproducibility measures and interventions in round 1

	Weighted average scores	% consensus^*^
**Reproducibility measure**		
Methodological quality	9.03	89.0%
Reporting quality	9.00	87.7%
Code and data availability and reuse	8.66	84.9%
Computational reproducibility	8.52	82.0%
Transparency of research plan	8.47	79.2%
Reproducible workflow practices	8.34	75.5%
Trial registration	8.21	74.5%
Materials availability and reuse	8.16	71.2%
**Intervention**		
Data management training	8.52	82.9%
Data quality checks/feedback	8.33	81.5%
Statistical training	8.33	80.2%
Data sharing policy/guidelines	8.22	77.6%
Protocol/trial registration	8.15	75.6%
Reproducible code/analysis training	8.11	73.8%

^*^Weighted averages represent mean scores across panellists. % consensus indicates the proportion of panellists assigning scores of 8–10 on a 10-point Likert scale

### Qualitative synthesis

The participants repeatedly applied similar reasoning when evaluating both reproducibility measures and interventions, and their qualitative feedback was presented together. Many comments addressed feasibility, disciplinary applicability, and the expected impact on reproducibility, regardless of whether an item was a measure or intervention. Interventions were often viewed as practical support for implementing the measures, whereas measures representing the standards that those interventions were intended to strengthen.

#### Round 1

In round 1, their comments consistently indicated that highly rated items were regarded as foundational and achievable within current research systems. Items such as *methodological* quality (score 9.03; consensus 89%) and *reporting quality* (score 9.00; consensus 87.7%) were frequently described as essential requirements for reproducibility and as areas where improvement was both feasible and measurable:

“This is (together with Methodological quality) the straightforward, number one reproducibility enabler!” – participant 24 (P24)

“This seems all encompassing and fundamental to good research practice.” – P54

Similarly, *code and data availability* (score 8.66; consensus 84.9%) and *material availability and re-use* (score 8.16; consensus 71.2%) were valued for promoting transparency and verification:

“This is probably one of the most relevant measures, since it allows for an effortless and error-free, interpretation-free reproduction.” – P26

“Yes, making the code available is very important, not only for validation but also to build upon what has already been done.” – P58

However, many panellists cautiously warned that this is not possible in all disciplines and case scenarios.

“It seems very important with the caveat that not everything can be made openly available (as open as possible, as protected as needed).”- P72

Furthermore, interventions emphasising training, such as *data management* (score 8.52; consensus 75.3%) and *statistical training* (score 8.33; consensus 71.23%), were considered practical mechanisms to strengthen these priorities:

“Data is the lifeblood and critical output of much research, and researchers must understand how to manage it.”- P43

“I think appropriate training for researchers could go a very long way towards high quality and reproducible research, I have given training initiatives the highest priority. “- P42

On the other hand, several measures and interventions with moderate-to-high weighted averages did not reach the ≥ 70% consensus threshold, reflecting conflicting views on feasibility and implementation rather than a lack of perceived importance. For example, *data access policies* (score of 7.95; consensus of 67.1%) and *computational reproducibility checks* (score of 7.50; consensus of 60.3%) were frequently acknowledged as important but associated with high resource demands and uncertain application:

“Such guidelines would be fantastic, but it would be important that they are harmonised across institutions and are in compliance with GDPR.” – P43

“This assumes that all researchers have access to the computational software and hardware required - this may not always be the case.” - P43

The participants also questioned the practicality of large-scale technical solutions, such as automated reproducibility platforms, and expressed concern about added administrative burden:

“Solutions like these respond to different needs and different customers, a one size fits all approach could complicate usability instead of promoting reproducibility.” – P61

The comments repeatedly noted disciplinary variability, emphasising that feasibility depends on data sensitivity, methodological norms, and existing infrastructure. The panellists noted that data protection and ethical restrictions in some fields limit the magnitude of sharing possible, whereas uneven access to computational tools and data-management infrastructure constrains implementation across disciplines. Also, several studies have suggested the importance of additional specialised statistical or data-support roles to make such measures viable. Overall, round 1 established a consensus around measures and interventions seen as both foundational and feasible. Items requiring substantial infrastructural change or external administration generated mixed responses and were therefore revisited in round 2 for further consideration, for a total of 6 reproducibility measures and 21 reproducibility interventions (see additional file 2).

#### Round 2

In round 2, the participants’ comments showed a shift from proposing new ideas to refining or confirming earlier views. Their feedback continued to emphasise the importance of feasibility and contextual variability in determining whether theoretically valuable actions could realistically enhance reproducibility.

Items focusing on policy or procedural transparency, such as the *transparency of funding sources* and the *transparency of interests and conflicts*, remained valued for promoting integrity but were considered only indirectly related to reproducibility outcomes:

“While transparency of funding may not directly influence whether a study can be reproduced, its role in fostering trust, reducing conflicts of interest, and upholding ethical standards makes it indispensable...” – P65

“Being honest about the transparency of interest is also very important, giving details around conflicts of interest (COIs), such as financial or political influences, is important for ethical research practices. These would affect research practices or process in some ways.” – P48

Similarly, the *transparency of contributions* was appreciated for clarifying author roles yet was seen primarily as a matter of fairness rather than reproducibility:

“I agree that while this is very important for fair attribution and trust, it doesn't directly impact the reproducibility of a study.” – P18

However, some panellists have argued the importance of this item for reproducibility as well:

“…One connection is to clearly identify how each author contributed to the research so that other researchers know who to contact when/if they attempt to replicate/reproduce the results.” – P63

Technically oriented measures, including *computational reproducibility checks* and *documentation systems*, continue to attract support because their potential to be consistently checked but face continuing feasibility concerns. The participants cited disciplinary differences in available software, infrastructure, and technical expertise, favouring round 1 feedback:

“Possibly - but this technology is not widely available and has not yet been adopted as standard practice across scientific fields.” – P63

However, several comments highlighted the feasibility of these items and their direct impact on reproducibility, although a general consensus was not reached:

“Documentation systems like electronic lab notebooks are vital for ensuring data integrity, transparency, and the long-term reliability of scientific research. While challenges such as accessibility and disciplinary fit exist... Their unparalleled ability to enhance research practices and support reproducibility makes them deserving of the highest priority - grade of 10.” – P65

Proposals centred on standardisation and recognition mechanisms, including interventions, such as *reporting checklists*, *registered reports*, and *open-access publishing*, agreed to promote transparency, but the panellists expressed reservations about uneven uptake across disciplines, the cost of open-access publication, and potential administrative burden:

“…While open-access (OA) publishing is helpful in making research more transparent and accessible globally, there are however concerns on high publication fees that are usually charged. There is also concern around the Article Processing Charges (APCs), which are charged…”- P48

“While still a relatively new concept, registered reports help reduce pressure to publish only positive results. However, the costs for researchers and peer review system need to be carefully considered.” – P18

Some responses overlapped with round 1 commentary, reinforcing a steady pattern of bias but not full endorsement. Some of the comments provided beneficial suggestions for future improvements:

“These can be game-changers for enforcing reporting standards. If journal/publisher-hosted AI tools automatically check, there is no danger that manuscripts will be fed to externally sourced AI tools of the big tech industry. I am confident most concerns can be circumvented, to make this a game-changing instrument in the complete reporting battle (one of the battlegrounds of the reproducibility war).” – P4

Other items, such as *mentoring and role-model programs* and *data-access policies or agreements,* continued to be perceived as valuable but lacked mechanisms for consistent implementation across institutions. Panellists emphasised that while such practices contribute to research culture, their impact on reproducibility depends on clear institutional responsibility and role division:

“The value of both role models (awards/prizes, ambassador programmes) AND one-to-one mentoring (which are two very different things) cannot be underestimated, also for reproducibility. Indeed, reverse mentoring (ECRs sharing good practices with their seniors) has great potential to change practices…” – P4

Overall, the round 2 feedback revealed solidifying rather than expanding of consensus. The panellists reaffirmed the priorities established in round 1, emphasising that progress towards reproducibility depends not only on methodological standards but also on structural capacity and institutional support.

#### Final consensus panel

In the final Delphi panel, eight panellists reviewed the outcomes of the previous rounds and confirmed the final priority lists. Two interventions, *data access policies/agreements* (score of 7.73) and *computational reproducibility checks/feedback* (score of 7.50), were reconsidered in the final round, as they were the highest-scoring items that had not reached consensus and were therefore closest to potential inclusion in the final list. After discussion, the participants agreed not to include the additional interventions and confirmed that the existing lists accurately reflected collective priorities. There was a consensus that all 8 reproducibility measures and 6 interventions that reached the priority list already in round 1 were important and that the ranking should be regarded as non-hierarchical, representing complementary aspects of reproducibility rather than a strict order of importance.

Discussion around the reproducibility measures focused primarily on refining the terminology and interpretation rather than changing the content or order of the items. The participants agreed to replace *“methodological quality”* with *“methodological robustness”* to better capture the intended meaning and to clarify *“reporting quality”* as *“reporting completeness and transparency”*:

“…Is it methodological robustness, as you say, you know, complete reporting, transparent reporting. I mean, obviously we want whatever is reported to be of quality. But is everything being reported? I suppose. So perhaps a little think about quality and how if there are maybe better words to really capture that…” – Speaker 5

“…there are lots of checklists for different types of studies and kind of existing guidelines. So I think it's much easier to define what like complete and detailed would mean, at least for the main types of studies.”- Speaker 7

There was shared recognition that these measures were interdependent and that their collective implementation was necessary to improve reproducibility, rather than any single measure serving as a stand-alone solution.

When discussing the interventions, the panel revisited *data-access policies or agreements* and *computational reproducibility checks and feedback*, both of which had ranked just below the consensus threshold in earlier rounds. The participants agreed that while these actions were conceptually valuable, their practical implementation varied widely across disciplines and institutions. It was therefore decided to acknowledge them as supportive contextual factors rather than to add them to the prioritised list:

“…I think it's better to have a maybe a shorter priority list, because if we have too many items, it may lead to like, you know, a bit of a decision paralysis if someone wants to prioritize some further points…”- Speaker 7

Broader discussion centred on balancing individual-level training interventions with structural or policy incentives, with several participants emphasising that reproducibility cannot depend solely on researcher effort without systemic support.

## Discussion

This Delphi study prioritised key measures and interventions to strengthen research reproducibility across disciplines. Through two iterative survey rounds and a final expert panel, consensus was achieved on eight reproducibility measures and six interventions, reflecting expert agreement on the most practical and foundational elements needed to improve reproducibility. Prioritised measures included methodological robustness, reporting completeness and transparency, code and data availability and reuse, computational reproducibility, transparency of research plan, reproducible workflow practices, trial registration, and materials availability and reuse; prioritised interventions included data management training, data quality checks/feedback, statistical training, data sharing policy/guidelines, protocol/trial registration, and reproducible code/analysis training. Methodological quality and transparent reporting, which came on the top of the priority list, have long been recognised as central pillars of reproducible research. There is a substantial body of evidence demonstrating how inadequate study design, incomplete methodological descriptions, and selective reporting undermine the ability to reproduce or validate research findings across disciplines [24]. Consistent with this literature, the prioritisation of methodological and reporting robustness, including trial registration, supports existing evidence that clear study design and transparent reporting are fundamental to improving reproducibility and reducing research waste [1, 2]. Our Delphi process extends prior work by explicitly prioritising these measures across disciplines and stakeholder groups.

### Prioritised measures and interventions

The prioritisation of transparency-related measures and their corresponding interventions, including trial or protocol registration and data and code availability, aligns with existing evidence emphasising transparency as a mechanism to reduce selective reporting and analytic cherry-picking [25]. Expert comments, however, nuanced this alignment by framing transparency foundational yet conditional. Data, code, and materials sharing were supported as reproducibility standards in principle, but their implementation was described as dependent on privacy, intellectual property, and feasibility considerations. Protocol or trial registration was prioritised as an important mechanism for operationalising transparency-related measures, particularly in clinical research, where systematic reviews and meta-analyses show associations with improved transparency alongside persistent discrepancies between registered and published outcomes [26]. Experts view protocol and trial registration not as a standalone solution for reproducibility but as a conditional transparency tool whose value depends on context, registration quality, and follow-up scrutiny, revealing a gap between formal adoption and effective use. This highlights the need for future research to figure out how registration practices can be adopted across study designs and disciplines to best support reproducibility in practice.

Reproducible workflow practices and material availability were assessed as important, reflecting expert recognition of the importance of traceable procedural and computational approaches in supporting reproducibility. Structured workflows, including continuous analysis, containerisation, and workflow management systems, have been shown to improve transparency and the re-execution of computational research practices [27]. Although researchers are increasingly encouraged to share their data, workflows and materials, empirical evidence indicates that actual practices remain inconsistent across disciplines and academic roles. Simply making all the data available is not enough to ensure reproducibility, without detailed metadata and precise documentation [28]. In our study, expert comments similarly highlighted that reproducible workflow practices and material availability are essential, but their applicability is strongly context dependent. Also, the experts pointed out the difference between reproducibility for computational workflows and digital artefacts compared to physical materials, which may be sensitive or impractical to share. The prioritisation of these measures therefore reflects the recognition of their enabling role in reproducibility, contingent on documentation quality, disciplinary norms, and available infrastructure rather than uniform implementation.

The prioritised reproducibility interventions from our study represent practical approaches for supporting and operationalising them in research practice. Training-focused measures, including data management training, statistical training, and reproducible code/analysis training, were strongly prioritised. However, a recent scoping review indicated that although training practices are widely recommended, the empirical evidence evaluating their effectiveness remains limited [29]. The experts’ comments provided important context for this gap, emphasising that training was regarded not as a standard solution but as a foundational component that must be supported by appropriate expertise and infrastructure. They noted that training may be insufficient for complex analytical work without access to specialist roles, such as statisticians or data scientists, and that uptake may be limited when incentives and research culture do not support implementation.

Beyond training, experts emphasised that reproducibility also depends on robust data governance structures, particularly mechanisms for data quality checks and clear data-sharing guidance. The literature promotes these interventions as mechanisms to improve transparency, reduce avoidable errors, and support reuse [30]. However, expert feedback highlights several operational questions that are often underspecified in existing guidance, including the responsibility for providing data quality feedback, the appropriate stage for such checks, and how procedures should be adapted to different data types. With respect to data sharing guidance, the experts emphasised the need for harmonisation across institutions and alignment with legal and ethical requirements while also acknowledging justified exceptions, such as research involving sensitive data and proprietary datasets.

### Evaluation challenges and evidence gaps

Recent evidence highlights that the impact of reproducibility and open science interventions and measures is often difficult to identify via conventional evaluation approaches, particularly when outcomes are assessed indirectly or over short timeframes [31]. Rather than demonstrating reliable, measurable effects on reproducibility outcomes, many interventions are characterised by heterogeneous implementation, context-dependent uptake and dispersed routes of influence. This challenge has also been used to identify priorities in areas where empirical evidence remains fragmented or incomplete [9]. This underscores the need for future systematic and context-dependent evaluation of reproducibility practices.

Consistent with these observations, the evidence map from the iRISE project shows that empirical evaluations are clustered primarily around reporting quality and data availability [32], whereas several interventions prioritised by the Delphi panel, including training-based interventions, reproducible workflow practices, and trial registration, remain sparsely evaluated across disciplines. Most evidence is also concentrated within biomedical research and social and health sciences, with limited assessment in other research areas. This means that, overall, limited or inconsistent empirical effects do not reflect a lack of importance but rather gaps in evaluation, disciplinary coverage, and methodological approaches to measuring impact.

### Strengths and limitations

Our study has several strengths and limitations. The Delphi process itself demonstrated sustained engagement once panellists entered the study, as reflected in high retention across rounds, which is consistent with good practices reported in the literature [33]. While the overall response rate relative to the number of invited experts was modest, the inclusion of multiple stakeholder perspectives, including researchers, editors, funders, publishers, and policymakers, ensured that diverse viewpoints informed the final lists. In the context of a Delphi study, such heterogeneity supports the relevance of the findings across different parts of the research system, although it does not eliminate differences in disciplinary norms or priorities [34]. However, discipline-specific bias cannot be fully mitigated, as reproducibility initiatives at this time receive greater interest in some fields than others do [35], despite our efforts to include panellists from a wide range of disciplines. Although participation remained high across two rounds, the small attrition between rounds may have affected representation, even if retention remained within acceptable ranges for Delphi studies [33] The stakeholder group distribution among participants was not systematically recorded, as the study prioritised cross-disciplinary expertise over formal role categorisation. This represents a limitation in terms of reporting the balance of stakeholder perspectives across rounds. The final consensus panel comprised eight participants representing all five stakeholder groups. While the distribution of panel members reflected the natural representation of roles within the research ecosystem, with researchers, editors, and publishers being more prevalent than.

funders and policymakers, the panel can be considered broadly representative of the expert community engaged in research reproducibility.

## Conclusions

The reproducibility measures and interventions prioritised in our study provide a structured foundation for future work to enhance reproducibility practices. By integrating perspectives from multiple stakeholder groups, the findings offer a focused set of priorities that can inform institutional policies, training curricula, and assessment frameworks. These results will be used for the future work of the research team, as we will conduct focus groups on facilitators and barriers to the implementation of these practices. These findings provide a pragmatic starting point for implementation while highlighting the need for empirical evaluation of reproducibility measures and interventions across research contexts.

## Supplementary Information

Additional file 1: Items included in Delphi round 1. Description: List and definitions of reproducibility measures and interventions evaluated in the Delphi study.

Additional file 2: Reproducibility measures and interventions revisited in the second Delphi round. Description: Items that did not reach consensus in round 1, together with their weighted average scores and percentage consensus.

Additional file 3. CREDES checklist.

## Data Availability

The datasets generated and/or analysed during the current study are not publicly available due to ethical restrictions and data protection requirements (GDPR), as they contain anonymised expert ratings and qualitative comments collected under conditions of confidentiality. The data are available from the corresponding author upon request. The study protocol is publicly available on the Open Science Framework (OSF).
