# Supplementary material for: Prioritising measures and interventions to strengthen research reproducibility: a Delphi consultation study
Source: Res Integr Peer Rev. 2026 Jul 3;11:31. doi: 10.1186/s41073-026-00217-y (PMC13330243; doi:10.1186/s41073-026-00217-y)
Supplement: Supplementary file 1 — Additional file 1: Items included in Delphi round 1. Description: List and definitions of reproducibility measures and interventions evaluated in the Delphi study. [file 41073_2026_217_MOESM1_ESM.docx]

**Prioritising interventions and reproducibility measures to improve research reproducibility: a Delphi consultation method**

Pejdo D, Buljan I, Marušić A.

**Additional file 1.** Items included in Delphi round 1

| **Reproducibility measures from SOLES (n=14)** |
| --- |
| Code and data availability and re-use - *Making data and code used in a study openly available and might be reused by others to verify or expand the study.* |
| Computational reproducibility - *The capacity of other researchers to accurately reproduce a study's results using the original data, code, and computational tools.* |
| Materials availability and re-use - *Ensuring that any digital or physical resources used in the study are accessible for other researchers to reproduce the results or expand the study.* |
| Methodological quality - *The extent to which a research study is designed, conducted, and reported to minimize systematic errors (bias) and ensure the validity and reliability of its findings.* |
| Transparency of research plan - *Open and clear documentation of a research study’s methodology, design, data collection, analysis, and intended outcomes before the study is conducted* |
| Absence of publication bias - *Selective publication of research studies based on their results, where studies with statistically significant positive findings are more likely to be published than those with non-significant or negative findings.* |
| Reporting quality - *The transparent and accurate presentation of research findings, methods, and results in a report publication.* |
| Reproducible workflow practices - *A set of principles, methods, and tools that enable researchers and scientists to design, execute, and share their computational workflows to ensure reproducibility, reusability, and transparency.* |
| Transparency of contributions - *The practice of clearly and openly disclosing the roles and responsibilities of each author or contributor in a research study.* |
| Transparency of evaluation - *The openness and clarity with which researchers assess other researchers' methods, procedures, and findings.* |
| Transparency of funding - *Open and clear disclosure of financial information, such as the sources, uses, and management funds.* |
| Transparency of interest - *Declaring any possible personal or institutional interests that may affect the study or its interpretation.* |
| Trial registration - *Pre-registering clinical or experimental studies encourages transparency and responsibility in research design and outcomes.* |
| Type I/II error reduction - *Lowering the chances of mistakenly claiming a result exists when it doesn’t (false positive) and missing a real result when it exists (false negative) to ensure more accurate research findings* |
| **Interventions from SOLES (n= 27)** |
| Reporting guidelines and checklists - *Tools ensuring full and consistent reporting of research methodologies and findings.* |
| Blockchain technology - *Database mechanism that allows safe, transparent, and tamper-proof data sharing and transactions.* |
| Centralized sharing platform - *A digital platform where researchers may upload, access, and exchange data, code, or resources in a single location.* |
| Code quality checks/feedback - *Process for reviewing and providing input on the accuracy, efficiency, and readability of software code.* |
| Code sharing policy/guidelines - *Rules or advice on how to distribute and maintain research code for reuse.* |
| Computational reproducibility checks/feedback - *Processes that ensure scientific computational analysis can be reliably reproduced and validated by others.* |
| Data access policies/agreements - *Regulations or agreements outlining the rules and conditions for accessing and using research data.* |
| Data quality checks/feedback - *Processes that ensure the data being collected, stored, and analyzed meets certain standards and is free from inconsistencies.* |
| Data sharing policy/guideline - *Frameworks on how and when research data should be made accessible for others to access and re-use.* |
| Data sharing statements - *Declarations by researchers on how they intend to make their data public, including any restrictions.* |
| Data management training - *Education and guidance on how to appropriately organize, store, and manage research data throughout its life cycle.* |
| Documentation system (e.g. electronic lab notebooks) - *An organized approach for recording and tracking all details about research methodologies, data, and code to ensure transparency.* |
| Materials sharing policy/guidelines - *Rules or recommendations for sharing research resources (such as reagents and samples) with others for replication or further research.* |
| Mentoring/role model - *Experienced researchers provide guidance to assist early career researchers build their professional and research abilities.* |
| Online sharing platform - *A web-based platform for sharing and disseminating research data, code, or discoveries with a large audience.* |
| Open-access publication - *A publishing approach in which research publications are freely available to the public without any subscription requirements.* |
| Open Science tools (Open Code badges, Open Data badges, Open Data Commons For Spinal Cord Injury, Open Materials badges, OpenStats, Open Science Framework, Open Science Plans) - *To encourage openness and cooperation, digital tools and platforms allow for the open sharing of research methodologies, data, and outcomes.* |
| Preregistration badges - *Researchers receive awards for openly publishing their study's research design and hypotheses before executing the experiment.* |
| Protocol/trial registration - *The procedure of formally submitting and recording the specifics of a research plan before conducting the study. For example:* [*ClinicalTrials.gov*](http://clinicaltrials.gov), [EU Clinical Trials Register](https://www.clinicaltrialsregister.eu/). |
| Registered reports- *A publication format in which the study design and procedures are peer-reviewed before data collection, reducing bias.* |
| Reporting quality checks/feedback (e.g. Penelope.ai) - *Tools used to assess the correctness, clarity, and accuracy of how research procedures and conclusions are published.* |
| Reproducible code/analysis training - *Education on the best techniques for developing and organizing code, as well as reproducible analysis.* |
| Reproducible coding environment - *A standardized computing configuration or environment that provides consistent code execution across several platforms.* |
| Choice of statistical plan (e.g. high-powered studies, multi-center setting, conservative p-values for significance) - *Techniques and procedures for collecting, analyzing, interpreting, and presenting quantitative data in research.* |
| Statistical training - *Education focused on training researchers how to properly analyze and interpret quantitative data.* |
| Critical assessment training - C*arrying out a critical assessment of evidence synthesis, including techniques for gathering, evaluating, and synthesizing information from different studies* |
| Workflow standardization - *Developing uniform, well-documented research protocols to ensure reproducibility and efficiency throughout multiple studies.* |
