# Supplementary material for: Prioritising measures and interventions to strengthen research reproducibility: a Delphi consultation study
Source: Res Integr Peer Rev. 2026 Jul 3;11:31. doi: 10.1186/s41073-026-00217-y (PMC13330243; doi:10.1186/s41073-026-00217-y)
Supplement: Supplementary file 2 — Additional file 2: Reproducibility measures and interventions revisited in the second Delphi round. Description: Items that did not reach consensus in round 1, together with their weighted average scores and percentage consensus. [file 41073_2026_217_MOESM2_ESM.docx]

**Prioritising interventions and reproducibility measures to improve research reproducibility: a Delphi consultation method**

Pejdo D, Buljan I, Marušić A.

**Additional file 2.** Reproducibility measures and reproducibility interventions that were revisited in the second round with their scores from round 1**.**

| Reproducibility measures scores (n=6): |
| --- |
| Type I/II error reduction (6.88) – 39.39 % |
| Transparency of evaluation (6.79) – 49.93% |
| Absence of publication bias (6.59) – 39.39 % |
| Transparency of interest (6.08) – 33.33% |
| Transparency of contributions (5.79) – 25.76% |
| Reproducibility intervention scores (n=21): |
| Data access policies/agreements (7.73) – 66.67 % |
| Computational reproducibility checks/feedback (7.50) – 57.58% |
| Reporting guidelines and checklists (7.38) – 54.54% |
| Choice of statistical plan (7.36) – 53.03% |
| Mentoring/role modelling (7.29) – 57.57% |
| Registered reports (7.12) – 48.48 % |
| Code sharing policy/guidelines (7.08) – 48.48 % |
| Data sharing statements (7.05) – 45.45% |
| Documentation system (7.00) – 50.00 % |
| Materials sharing policy/guidelines (7.00) – 54.54% |
| Critical assessment training (6.89) – 43.94 % |
| Code quality checks/feedback (6.77) – 45.45% |
| Reporting quality checks/feedback (6.70) – 39.39% |
| Reproducible coding environment (6.64) – 37.88 % |
| Online sharing platform (6.48) – 34.85 % |
| Workflow standardisation (6.48) – 36.36 % |
| Open-access publication (6.32) - 34.85 % |
| Open Science tools (6.08) – 31.82 % |
| Centralised sharing platform (5.14) – 16.67 % |
| Preregistration badges (5.02) – 10.60% |
| Blockchain technology (4.38) – 6.06 % |
