# Supplementary material for: Prioritising measures and interventions to strengthen research reproducibility: a Delphi consultation study
Source: Res Integr Peer Rev. 2026 Jul 3;11:31. doi: 10.1186/s41073-026-00217-y (PMC13330243; doi:10.1186/s41073-026-00217-y)
Supplement: Supplementary file 3 — Additional file 3. CREDES checklist. [file 41073_2026_217_MOESM3_ESM.docx]

**Prioritising interventions and reproducibility measures to improve research reproducibility: a Delphi consultation method**

Pejdo D, Buljan I, Marušić A.

**Additional file 3**. CREDES checklist

| **CREDES domain** | **CREDES item** | **Description (CREDES recommendation)** | **Where addressed in manuscript (page/section)** |
| --- | --- | --- | --- |
| **Rationale** | Justification for Delphi | Clear rationale for using the Delphi method rather than alternative consensus methods | p. 6 |
| **Planning and design** | Study aim | Clear statement of the aim(s) of the Delphi study | p. 2 & 6 |
|  | Definition of consensus | A priori definition of how consensus was defined and measured | Table 1 & p. 9 |
|  | Panel eligibility criteria | Explicit eligibility criteria for expert panellists | Table 2. & p. 5 |
|  | Panel composition | Description of the professional background and expertise of panellists | Table 2. & p.5 |
|  | Recruitment strategy | Description of how panellists were identified, invited, and recruited | p. 5 |
|  | Sample size | Justification or explanation of the panel size | p. 5 |
|  | Number of rounds | Planned number of Delphi rounds and rationale | p. 6 & 7 |
|  | Questionnaire development | Description of how Delphi items were generated or selected | p. 3 |
|  | Pilot testing | Whether the questionnaire was piloted and how | / |
| **Study conduct** | Anonymity | Measures taken to ensure anonymity of panellists during rating | p. 5 |
|  | Data collection method | Mode of data collection (e.g. online survey, email, meeting) | p. 4 |
|  | Feedback between rounds | Type and content of feedback provided to panellists between rounds | p. 6 & 7 |
|  | Controlled feedback | Explanation of how feedback was standardised and controlled | p. 7 - 9 |
|  | Attrition | Reporting of response rates and dropouts for each round | p. 9 & 10 |
|  | Stopping criteria | Criteria used to determine when the Delphi process was stopped | / |
|  | Deviations from protocol | Description and justification of any deviations from the original protocol | / |
| **Analysis** | Quantitative analysis | Methods used to analyse quantitative ratings | p. 10 |
|  | Qualitative analysis | Methods used to analyse open-ended responses or comments | p. 10 |
|  | Handling of disagreement | Description of how lack of consensus or disagreement was handled | / |
| **Reporting** | Presentation of results | Clear presentation of items reaching and not reaching consensus | Table 2 |
|  | Transparency of process | Sufficient detail to allow replication of the Delphi process | Throughout the manuscript |
|  | Limitations | Discussion of methodological limitations of the Delphi study | p. 24 |
